# Association between RANTES/CCL5 levels with *Plasmodium* infections and malaria severity: a systematic review

**DOI:** 10.1186/s12936-024-05152-1

**Published:** 2024-11-09

**Authors:** Pattamaporn Kwankaew, Aongart Mahittikorn, Wanida Mala, Kwuntida Uthaisar Kotepui, Nsoh Godwin Anabire, Polrat Wilairatana, Manas Kotepui

**Affiliations:** 1 Medical Technology, School of Allied Health Sciences, Walailak University, Tha Sala, Nakhon Si Thammarat, Thailand; 2 Department of Protozoology, Faculty of Tropical Medicine, Mahidol University, Bangkok, 10400 Thailand; 3 Medical Technology Program, Faculty of Science, Nakhon Phanom University, Nakhon Phanom, Thailand; 4 Department of Biochemistry & Molecular Medicine, School of Medicine, University for Development Studies, Tamale, Ghana; 5 West African Centre for Cell Biology of Infectious Pathogens (WACCBIP), Department of Biochemistry, Cell & Molecular Biology, University of Ghana, Accra, Ghana; 6 Department of Clinical Tropical Medicine, Faculty of Tropical Medicine, Mahidol University, Bangkok, Thailand

**Keywords:** *Plasmodium*, Malaria, RANTES, CCL5, Systematic review

## Abstract

**Background:**

Malaria continues to be a significant global health concern, and developing effective therapeutic strategies requires an understanding of the immune response to the disease. This systematic review synthesized the current body of research on the role of regulated on activation, normal T cell expressed and secreted (RANTES)—in the pathogenesis and disease severity of malaria.

**Methods:**

A systematic review protocol was registered with PROSPERO under the registration number CRD42024535822. The systematic review was conducted following PRISMA guidelines to identify studies examining RANTES levels in individuals infected with *Plasmodium* species. Searches were performed across multiple databases, including ProQuest, Journals@Ovid, Embase, Scopus, PubMed, and MEDLINE. Further searches were performed in Google Scholar. Quality assessment was done using the Joanna Briggs Institute (JBI) critical appraisal tools. Alterations in RANTES levels in patients with malaria were synthesized narratively.

**Results:**

A comprehensive search of major databases identified 22 studies meeting inclusion criteria, predominantly focusing on *Plasmodium falciparum* and *Plasmodium vivax* infections. RANTES levels were found to vary significantly across different severities of malaria, with several studies reporting lower levels in severe cases compared to non-malarial controls. However, inconsistencies were observed in the alterations of RANTES levels between severe and non-severe malaria cases.

**Conclusion:**

Taken together, the finding of this systematic review underscore the complex regulation of RANTES in malaria pathophysiology. Future research should focus on longitudinal assessments to elucidate the dynamic role of RANTES throughout the course of malaria and recovery, to potentially inform the design of novel therapeutic strategies.

**Supplementary Information:**

The online version contains supplementary material available at 10.1186/s12936-024-05152-1.

## Background

Malaria is a disease prevalent in tropical and subtropical regions of the world, with an estimated 249 million cases in 85 endemic countries and areas in 2022 [1]. It is caused by parasitic *Plasmodium* species, most commonly *Plasmodium falciparum*, and is transmitted through the bites of infected female *Anopheles* mosquitoes [2]. Besides *P. falciparum*, malaria can be caused by *Plasmodium vivax*, *Plasmodium ovale wallikeri, Plasmodium ovale curtisi, Plasmodium malariae*, and *Plasmodium knowlesi* [3–5]. The disease can range from mild acute febrile episodes to severe complications like cerebral malaria, which involves multi-organ damage and is particularly deadly [6, 7]. Globally, malaria caused an estimated 608,000 deaths in 2022, with most occurring in children under 5 years of age [1].

The immune response to malaria is complex and involves both innate and adaptive immunity [8, 9]. Upon infection, the body’s immediate defense mechanisms include the activation of macrophages and the release of pro-inflammatory cytokines, which help control the early stages of the parasite [10, 11]. Regulatory cytokines and chemokines, involved in leukocyte trafficking and activation, play a crucial role in controlling parasitemia and eliminating infection. Key cytokines and chemokines include interferon-gamma (IFN-γ), tumour necrosis factor (TNF), interleukin (IL)-10, IL-17, IL-4, and the regulated on activation, normal T cell expressed and secreted (RANTES) [12–18].

RANTES, also known as C–C motif chemokine ligand 5 (CCL5), is a 68-amino acid chemokine involved in orchestrating the immune response, playing a significant role in the recruitment and activation of various immune cells during an immune challenge [19]. RANTES facilitates the trafficking and homing of classical lymphoid cells by binding to its receptor [19]. This pro-inflammatory chemokine, predominantly generated by CD8 + T cells, fibroblasts, epithelial cells, and platelets, is a hallmark of inflammation. Increased RANTES expression has been linked to various inflammatory disorders and pathologies, including allogeneic transplant rejection, atherosclerosis, arthritis, atopic dermatitis, and other inflammatory conditions [20]. RANTES promote leukocyte migration by binding to receptors in the seven-transmembrane G protein-coupled receptor (GPCR) family, including C-C motif chemokine receptor (CCR) 1, CCR3, CCR4, and CCR5 [21]. It promotes the infiltration of leukocytes (such as T cells and monocytes, basophils, eosinophils, natural killer cells, dendritic cells, and mast cells) to sites of inflammation [20, 22].

Prior studies have shown that differences in the RANTES gene affect the synthesis of the RANTES protein and the host's ability to fight against different infections [23, 24]. In the context of malaria, low amounts of RANTES protein have been observed in cases of severe malaria, which may be related to thrombocytopenia brought on by malaria or monocytes acquiring *Plasmodium* haemozoin [15]. Children with cerebral malaria have a higher death rate when their RANTES levels are lower [25]. Although RANTES levels were reported to be lower in patients with malaria, the precise role of RANTES in *Plasmodium* infection in relation to severity remains unclear. This systematic review aims to collate evidence of RANTES levels in individuals infected with *Plasmodium* species. Understanding the differences in this key chemokine and the resulting immunomodulation may provide crucial insights into malaria pathology.

## Methods

### Protocol and registration

This systematic review protocol was registered with PROSPERO under the registration number CRD42024535822. The reports follow the Preferred Reporting Items for Systematic Reviews and Meta-Analyses (PRISMA) guidelines [26].

### Search strategy

A systematic review was conducted to identify studies that examined the levels of RANTES in patients infected with *Plasmodium* species. The search involved several databases, including ProQuest, Journals@Ovid, Embase, Scopus, PubMed, and MEDLINE. The search terms used were: “(RANTES OR CCL5 OR “RANTES Protein” OR “T-Cell RANTES Protein” OR “CCL5 Chemokine”) AND (malaria OR plasmodium OR “*Plasmodium* Infection” OR “Remittent Fever” OR “Marsh Fever “ OR Paludism)” (Table S1). There were no restrictions on the language or publication date of the retrieved articles. Additionally, Google Scholar was searched to ensure all relevant articles were included.

### Eligibility criteria

The inclusion criteria for the studies were as follows: studies involving human participants infected with *Plasmodium* species, studies measuring RANTES levels in plasma or serum, and studies comparing RANTES levels in malaria patients to non-malarial controls or between different severities of malaria. The exclusion criteria included animal or in vitro studies, conference abstracts without full-text articles, studies lacking relevant information on RANTES, or non-original articles.

### Study selection and data extraction

Initially, records were retrieved and identified from databases and imported to EndNote software (Version 20, Clarivate Analytics, UK). After removing duplicates, the remaining records were screened based on predetermined inclusion and exclusion criteria, focusing on studies that investigated RANTES levels in infected and uninfected individuals and the association of RANTES with malaria severity. Study selection was independently performed by two authors (MK, AM), with discrepancies resolved by consensus.

The following data were extracted from each study: author and year of publication; continent and country; study design; year of experiments; number and characteristics of participants; *Plasmodium* species; age range; RANTES levels in patients infected with *Plasmodium* species (and also non-malarial controls); RANTES levels in quantitative values (mean ± standard deviation or median with range); parasite density; method for detection of *Plasmodium* parasites; and method for RANTES quantification. Data extraction was performed independently by two authors (MK, AM). Discrepancies were resolved by consensus or consultation with a third author (PK).

### Risk of bias assessment and data syntheses

The risk of bias among the included studies was assessed using the Joanna Briggs Institute (JBI) tool, a critical appraisal tool for evaluating the methodological quality of various study designs, including case–control, cross-sectional, randomized controlled trials (RCTs), and cohort studies [27]. For case–control studies, the JBI tool evaluates factors like comparability of cases and controls, consistency in criteria application, exposure measurement reliability, and handling of confounding factors. For cross-sectional studies, it focuses on inclusion criteria clarity, subject description, measurement validity and reliability, and appropriate statistical analysis. For RCTs, the tool assesses randomization, blinding, follow-up completeness, and the rigour of outcome measurement. Lastly, for cohort studies, it examines the identification and management of confounding factors, follow-up completeness, exposure measurement validity, and the appropriateness of statistical methods used. A narrative synthesis using a thematic synthesis approach was applied to synthesize the findings of the reviewed studies [28]. A meta-analysis was not conducted due to the inadequate quantitative data on RANTES levels in participants with *Plasmodium* infections and comparison groups.

## Results

### Search results

**Fig. 1:**
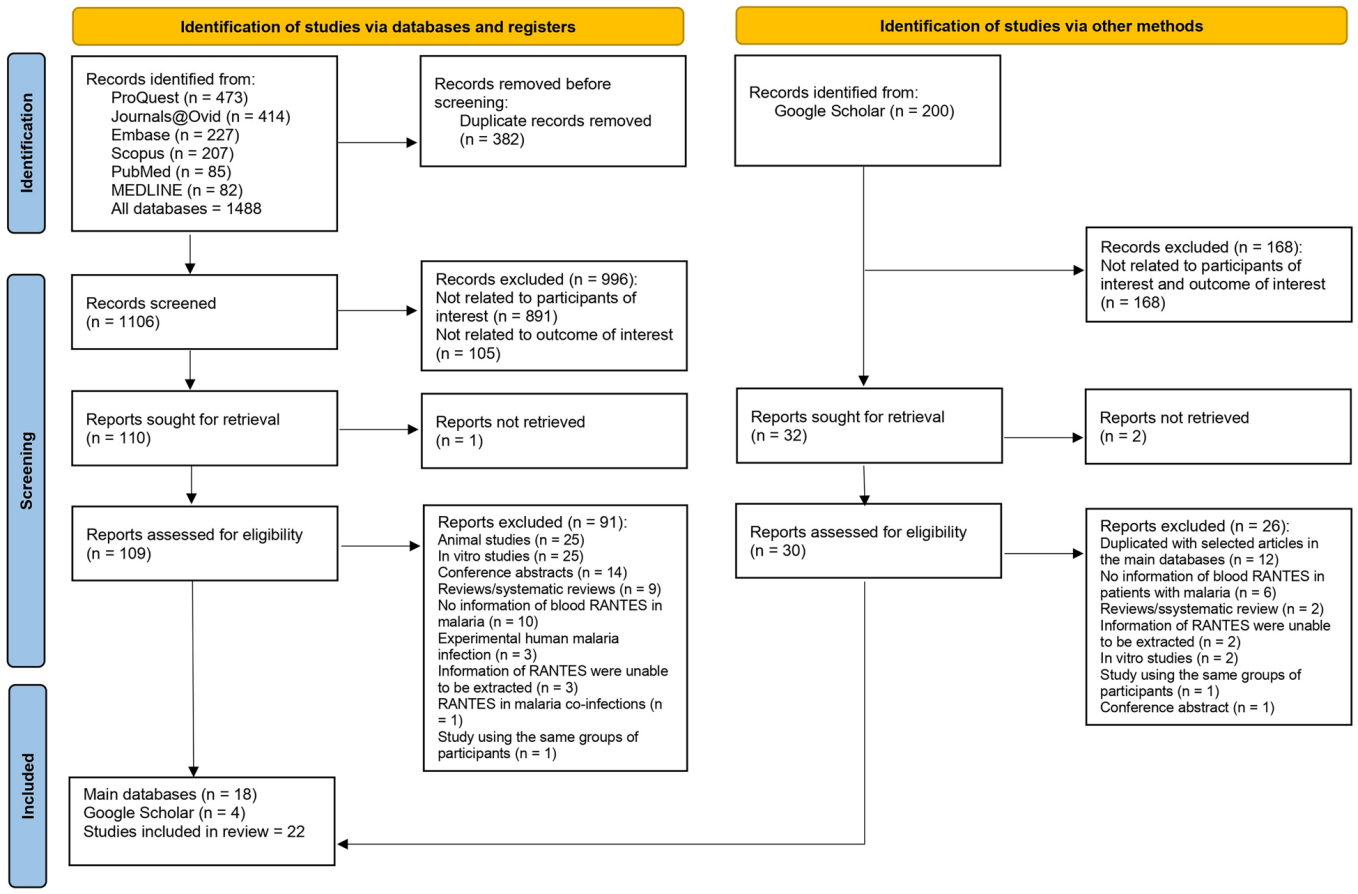
Study flow diagram

Initially, 1,488 records were identified from main databases such as ProQuest, Journals@Ovid, Embase, Scopus, PubMed, and MEDLINE, with 382 duplicates removed. Of the remaining records, 996 were excluded for not meeting the criteria related to participants or outcomes. Consequently, 110 reports were sought for retrieval, but 1 report was not retrieved. A total of 109 reports were assessed for eligibility, with 91 being excluded for reasons such as being animal or in vitro studies, conference abstracts, or lacking relevant RANTES information. An additional 200 records were identified via Google Scholar. Of these records, 32 reports were sought, 2 were not retrieved, and 30 were assessed, leading to 26 exclusions for specific reasons that they did not meet the inclusion criteria. Finally, 22 studies were included in the review [11, 15, 29–48]: 18 from the main databases [11, 15, 30–36, 38–43, 46–48] and 4 from Google Scholar [29, 37, 44, 45] (Fig. 1).

### Characteristics of included studies

The included studies in the systematic review predominantly focus on *P. falciparum* infections, accounting for 86.36% of the studies, with a smaller portion (13.64%) examining *P. vivax* (Table 1). Most of these studies (54.55%) were published between 2010 and 2019, while 27.27% were published between 2000 and 2009, and 18.18% from 2020 to 2023. In terms of study design, the majority were case–control studies (40.91%), followed by cross-sectional (27.27%) and cohort studies (27.27%), with only one randomized controlled trial (4.55%). Geographically, the studies were primarily conducted in Africa (72.73%), notably in Ghana, Kenya, Mali and Uganda. Asia and South America also contributed to the dataset, with India and Brazil each accounting for 13.64% of the studies conducted. Participants were predominantly children (54.55%), with studies also including adults (18.18%), both children and adults (18.18%), and pregnant women (9.09%). Microscopy was the most common malaria detection method (50%), often supplemented by polymerase chain reaction (PCR) (18.18%) or rapid diagnostic tests (RDTs) (22.73%). For RANTES detection, bead-based assays were used in the majority of studies (72.73%), while ELISA was employed in 22.73% of the studies. With regards to the type of blood sample used, RANTES measurements were predominantly from plasma (86.36%), with a minority using serum (13.64%).

**Table 1:** Summary characteristics of included studies

Characteristics	n. (22 studies)	%
Publication year		
2000–2009	6	27.27
2010–2019	12	54.55
2020–2023	4	18.18
Study designs		
Case–control study	9	40.91
Cross-sectional study	6	27.27
Cohort study	6	27.27
Randomized controlled trial	1	4.55
Study areas		
Asia	3	13.64
India	3	13.64
Africa	16	72.73
Ghana	3	13.64
Kenya	3	13.64
Mali	2	9.09
Uganda	2	9.09
Rwanda	1	4.55
Mozambique	1	4.55
Gabon	1	4.55
Nigeria	1	4.55
Tanzania	1	4.55
Cameroon	1	4.55
South America	3	13.64
Brazil	3	13.64
*Plasmodium* species		
*P. falciparum*	19	86.36
*P. vivax*	3	13.64
Participants		
Children	12	54.55
Adults	4	18.18
Children and adults	4	18.18
Pregnant women	2	9.09
Methods for malaria		
Microscopic method	11	50.00
Microscopic method/PCR	4	18.18
Microscopic method/RDT	5	22.73
PCR	1	4.55
Not specified	1	4.55
Assays for RANTES		
Bead-based assay	16	72.73
ELISA	5	22.73
Not specified	1	4.55
Blood samples		
Plasma	19	86.36
Serum	3	13.64

ELISA, Enzyme-linked immunosorbent assay; PCR, polymerase chain reaction; RDT, rapid diagnostic test

### Risk of bias assessment

For cross-sectional studies included in the review, Aguilar et al*.* [29] and Noone et al. [40] demonstrated the lowest risk of bias, meeting all criteria, including clear inclusion criteria, detailed subject description, valid and reliable measurements, and appropriate statistical analysis. Boström et al*.* [32], Panda et al. [43], and Turner et al*.* [46] showed some risk of bias due to inadequate identification and management of confounding factors, despite meeting most other criteria. Were et al. [48] exhibited the highest risk of bias, with unclear aspects such as exposure measurement, use of objective criteria, and statistical analysis.

For case–control studies included in this review, most studies ensured group comparability and proper matching of cases and controls, consistently applied criteria for identification, and used reliable exposure measurement methods. However, Frimpong et al*.* [37] did not consistently apply criteria for identifying cases and controls, and several studies either did not address or unclearly addressed confounding factors. For cohort studies included in the review, there were variations in the identification and handling of confounding factors and completeness of follow-up. Boström et al*.* [33] and Suguitan et al. [45] addressed confounding factors adequately but had issues with incomplete follow-up. Bujarbaruah et al*.* [34] and Ochiel et al*.* [41] did not identify or address confounding factors and had follow-up issues. Reuterswärd et al*.* [44] and Vinhaes et al*.* [47] identified confounding factors but did not comprehensively report on follow-up strategies. All studies used valid and reliable exposure measurement methods and applied appropriate statistical analyses (Table S2).

### RANTES levels in malaria compared to non-malaria patients

Table 2 presents details from the included studies, focusing on RANTES levels in patients with malaria. Seventeen studies reported comparisons of RANTES levels between patients with malaria and non-malarial controls [11, 15, 29–35, 37–39, 41, 44–46, 48]. Several studies consistently found significantly lower RANTES levels in severe malaria compared to non-malarial controls [15, 29, 31, 33, 34, 38, 41, 44, 48]. For instance, Boivin et al*.* observed lower RANTES levels in severe malaria compared to community controls in their randomized controlled trial [31], while Were et al. reported lower RANTES levels in severe malarial anaemia compared to healthy controls [48]. John et al. noted decreased RANTES levels in cerebral malaria compared to community controls [15], and Ochiel et al*.* found lower RANTES levels in children with both mild and severe malaria compared to healthy controls [41]. Bujarbaruah et al*.* reported significantly lower serum RANTES concentrations in severe malaria cases compared to control cases [34], and Frimpong et al*.* observed lower RANTES levels in children with malaria compared to children with sepsis but higher than febrile controls [37].

**Table 2:** Details of included studies

No	Authors	Study design	Study location (year)	Participants (n)	*Plasmodium* spp.	Age range	Qualitative results of RANTES	Parasite density	Method for malaria detection	Method for RANTES detection	Blood samples for RANTES
1	Aguilar et al., 2019 [29]	Cross-sectional study	Mozambique	Individuals of all ages: year 2010 (981), year 2013 (980)	*P. falciparum*	All age ranges	RANTES levels were lower in infected individuals compared to uninfected individuals	Infected (2010): 10.46 (1.61–102.94), Infected (2013):7.61 [1.43–299.84]	Microscopic method/PCR	Cytokine Human Magnetic 30-Plex Panel from Life Technologies™	Plasma
2	Armah et al., 2007 [30]	Case–control study	Ghana	Cerebral malaria (9), SMA (5), non-malaria deaths/non-malarial controls (5)	*P. falciparum*	Children	No difference in RANTES levels between children with cerebral malaria, SMA, and non-malarial controls	Cerebral malaria (9): 51,604 ± 9468, severe malarial anaemia (5): 195003 ± 23613, non-malaria deaths/non-malarial controls (5): 0	Microscopic method	Multiplex colorimetric bead-based cytokine immunoassay	Serum
3	Boivin et al., 2019 [31]	Randomized controlled trial	Uganda	The malaria survivor’s cohort of children (150): children with cerebral malaria (93), children with SMA (57)	*P. falciparum*	Children (6 to 12 years)	1. Severe malaria survivors with higher levels of plasma RANTES had better KABC cognitive performance after both titrating and non-titrating CCRT compared to no CCRT. For the CBCL, high plasma RANTES was associated with no benefit from either the titrating and non-titrating CCRT. 2. Control groups; RANTES levels were lower in severe malaria compared to community controls	Not specified	Microscopic method/RDT	Microbead suspension array technology (SAT) using the Luminex system (Austin, TX) and human-specific bead sets (R&D Systems, Minneapolis, MN)	Plasma
4	Boström et al., 2012 [a] [32]	Cross-sectional study	Mali	Uninfected Dogon (20), Infected Dogon (20), Uninfected Fulani (23), Infected Fulani (14)	*P. falciparum*	Children (2–10 years)	1. RANTES levels were significantly lower in infected Dogon compared to uninfected Dogon. 2. No difference in RANTES levels between infected Fulani compared to uninfected Fulani	Infected Dogon (20):13692 (575–48625), Infected Fulani (14): 27537 (100–122000)	Microscopic method	Cytometric bead array (CBA, BD Biosciences, San Diego, CA, USA)	Plasma
5	Boström et al., 2012 (b) [33]	Cohort study	Tanzania	Pregnant women with a gestational age ≤ 24 weeks (1000): malaria infected (42), malaria uninfected (79)	*P. falciparum*	Not specified	RANTES levels were significantly lower in infected individuals compared to uninfected individuals	Pregnancy women: malaria infected (11): 27,969.2 (39.5–390749 ± 17132.4)	Microscopic method/RDT	Cytometric bead arrays (CBA, BD Biosciences, San Diego, CA, USA)	Plasma
6	Bujarbaruah et al., 2017 [34]	Cohort study	India	Clinically proven *P. falciparum* malaria cases (153): uncomplicated malaria (128), severe malaria (25); age and sex matched community healthy controls without any past history of malaria infection (112)	*P. falciparum*	Adults	Serum RANTES concentrations was significantly lower in severe malaria cases [15708.92 ng/L] compared to uncomplicated malaria cases [16147.74 ng/L] and control cases [18587.2 ng/L]	Not specified	Microscopic method/RDT	Human RANTES ELISA kit (ab100633, Abcam)	Serum
7	Cruz et al., 2019 [35]	Case–control study	Brazil	Individuals from the Brazilian Amazon (601): symptomatic *P. vivax* monoinfected patients (179), asymptomatic *P. vivax* monoinfection (145), *P. vivax*-HBV coinfected patients (28), HBV monoinfected subjects (29), healthy controls (165)	*P. vivax*	Adults	No difference in RANTES levels between symptomatic vivax patients compared to uninfected controls	Symptomatic *P. vivax* monoinfected patients (179): 6324 (913.5–60623), asymptomatic *P. vivax* monoinfection (145): 0 (0–32), *P. vivax*-HBV coinfected patients (27): 753 (444.3–4262)	Microscopic method/PCR	Cytometric Bead Array—CBA (BD Biosciences Pharmingen, San Diego, CA, USA)	Plasma
8	Daveport et al., 2012 [36]	Case–control study	Kenya	Children aged 3–36 months with *P. falciparum* parasitemia (194): malaria alone (HIV-1( −)/Pf( +), n = 148); HIV-1 exposed (HIV-1(exp)/Pf( +), n = 30); and co-infected (HIV-1( +)/Pf( +), n = 16)	*P. falciparum*	3–36 months	RANTES levels were highest in co-infected individuals, high in individuals with malaria alone, and lowest in those exposed to HIV-1 and Pf( +)	Geometric mean; Malaria alone (HIV-1( −)/Pf( +), n = 148): 25,619 (59^,^721); HIV-1 exposed (HIV-1(exp)/Pf( +), n = 30): 25659 (35,865); and co-infected (HIV-1) +)/Pf( +), n = 16): 14405 (25599)	Microscopic method	Cytokine 25plex Antibody Bead Kit, Human [BioSource™ International]	Plasma
9	Frimpong et al., 2022 [37]	Case–control study	Ghana	Children (76): clinical malaria with no sepsis (33), non-malaria febrile control (20), non-malaria sepsis (23)	*P. falciparum*	Children	RANTES levels were significantly lower in children with sepsis when compared to children with malaria, but was higher when compared to febrile controls	Children (76): clinical malaria with no sepsis (32): 94494.87	Microscopic method/RDT	Human Cytokine Magnetic 25-Plex Panel (Thermo Fisher Scientific Corporation, United States)	Plasma
10	Hojo-Souza et al., 2017 [38]	Case–control study	Brazil	*P. vivax* uncomplicated patients (75), *P. vivax*-treated group (10), endemic control (10), healthy control (15)	*P. vivax*	Adults	1. RANTES levels were significantly lower individuals with *P. vivax* infection compared to endemic controls. 2. RANTES levels were significant increase after the treatment when following the same individuals	*P. vivax* uncomplicated patients (75): ≤ 500 (33), 501–10000 (25), 10001–100000 (8), without information (7)	Microscopic method/PCR	Cytometric bead assay (CBA) (BD Biosciences, USA)	Plasma
11	Jain et al., 2008 [39]	Case–control study	India	Cerebral malaria survivors (48), cerebral malaria non-survivors (12), healthy controls (25), mild malaria (48)	*P. falciparum*	Children (< 18 years) and adults (≥ 18 years)	No difference in RANTES levels between cerebral malaria survivors, cerebral malaria non-survivors, mild malaria, and healthy controls	Cerebral malaria survivors (46): 4166 ± 650.9, cerebral malaria non-survivors (12): 1336 ± 386.2, mild malaria (46): 1594 ± 426.6	Microscopic method	Multiplex bead-based cytokine immunoassay (MMA)	Plasma
12	John et al., 2006 [15]	Case–control study	Uganda	Children with cerebral malaria (88), children with uncomplicated malaria (76), community controls (100)	*P. falciparum*	4–12 years	1. RANTES levels were lower in cerebral malaria compared to uncomplicated malaria. 2.RANTES levels were lower in cerebral malaria compared to community controls. 3. RANTES levels at 72 h after admission were significantly higher than those at the time of admission but were comparable to those in children with uncomplicated malaria and were still not as high as those in community controls	Children with cerebral malaria (88): 39790 (143–560), children with uncomplicated malaria (76): 54840 (118–220)	Microscopic method	Colorimetric bead assay using the Luminex system and human-specific bead sets (R&D Systems)	Serum
13	Noone et al., 2013 [40]	Cross-sectional study	Nigeria	Uninfected endemic controls (69), *Ascaris* (21), Malaria (109), *Ascaris*/Malaria (32)	*P. falciparum*	39–73 months	RANTES was not associated with *P. falciparum* parasitemia	Malaria (109): 5097 ± 717.4, *Ascaris*/Malaria (31):6618.7 ± 1683.2	Microscopic method	DuoSet ELISA Developmental Kits (R&D, Minneapolis, MN, USA)	Plasma
14	Obeng-Aboagye et al., 2023 [11]	Case–control study	Ghana	Children (57); severe malaria (27), uncomplicated malaria (10), non-malaria related fever (20)	*P. falciparum*	Children	1. RANTES levels were significantly higher in severe malaria as compared to febrile controls. 2. No difference in RANTES levels between severe and uncomplicated malaria. 3. No difference in RANTES levels between uncomplicated malaria and febrile controls	Severe malaria (26): 54683 (29706–162776), uncomplicated malaria (10): 36228 (15671–116416)	Microscopic method	Human Cytokine Magnetic 25-Plex Panel (Thermo Fisher Scientific Corporation, United States of America)	Plasma
15	Ochiel et al., 2005 [41]	Cohort study	Gabon	Severe malaria cases (10), mild malaria cases (10), healthy malaria-exposed subjects (23)	*P. falciparum*	2 to 7 years	1. RANTES levels were significantly lower in children with mild malaria and severe malaria compared to healthy controls. 2. RANTES levels were significantly lower in children with severe malaria compared to those with mild malaria	Severe malaria cases (10): 355571 ± 58050, mild malaria cases (10): 52899 ± 10437	Microscopic method	Quantitative ELISA (Biosource International, Camarillo, CA)	Plasma
16	Ong’echa et al., 2011 [42]	Case–control study	Kenya	Uncomplicated malaria (31), non-SMA (37), SMA (80)	*P. falciparum*	Children (3 to 30 months)	No difference in RANTES levels between patients with SMA, non-SMA, and uncomplicated malaria	Uncomplicated malaria (30): 48354 (IQR 87430), non-SMA (36): 22615 (IQR 49,929), SMA (80): 26166 (IQR 60703)	Microscopic method	Human cytokine 25-plex antibody bead kit (BioSource International)	Plasma
17	Panda et al., 2013 [43]	Cross-sectional study	India	Severe malaria (125), non- complicated malaria (71), healthy controls (38)	*P. falciparum*	Severe malaria (125): 34(15–72), non- complicated malaria (71): 30(14–70), healthy controls (38): 30(12–75)	RANTES levels were significantly lower in severe malaria compared to non-complicated malaria	Not specified	Microscopic method/RDT	Commercial sandwich ELISA kits (Sanquin, Amsterdam)	Plasma
18	Reuterswärd et al., 2018 [44]	Cohort study	Rwanda	Severe malaria (180), mild malaria (183), controls (178)	*P. falciparum*	3 months up to 6 years	RANTES levels were significantly lower in infected individuals compared to uninfected individuals	Not specified	Microscopic method	Antibody-based suspension bead array	Plasma
19	Suguitan et al., 2003 [45]	Cohort study	Cameroon	Malaria positive (89), malaria negative (83)	*P. falciparum*	Pregnant women	No difference in RANTES levels between infected individuals compared to uninfected individuals	Placenta sample: 0.076 ± 0.154, Peripheral blood: 0.005 ± 0.011	Microscopic method	DuoSet ELISA Development System; R&D Systems	Plasma
20	Turner et al., 2021 [46]	Cross-sectional study	Mali	Asymptomatic infected with *P. falciparum* (8), uninfected subjects (27), children (19)	*P. falciparum*	Children and adults	Adults: No difference in RANTES levels between infected individuals compared to uninfected individuals	Not specified	PCR	Cytometric Bead Array Human Inflammatory Cytokine Kit (BD Biosciences)	Plasma
21	Vinhaes et al., 2021 [47]	Cohort study	Brazil	Asymptomatic malaria (108), symptomatic malaria (134); mild malaria (106), severe malaria (28), uninfected endemic controls (128)	*P. vivax*	Adults	RANTES levels were significantly higher in symptomatic malaria compared to asymptomatic malaria	Not specified	Microscopic method/PCR	Cytometric Bead Array—CBA (BD Biosciences Pharmingen, San Diego, CA, USA)	Plasma
22	Were et al., 2006 [48]	Cross-sectional study	Kenya	SMA (27), moderate anaemia (27), mild anaemia (28), healthy controls (24)	*P. falciparum*	Children (age < 36 months)	RANTES levels were decreased with increasing malarial anaemia severity, with the SMA group having lower circulating RANTES than children with moderate malarial anaemia, mild malarial anaemia, or healthy controls	SMA (26): 38874 ± 7685, moderate anaemia (26): 37771 ± 8169, mild anaemia (27): 34183 ± 7909	Not specified	Not specified	Plasma

*CCRT* Computerized cognitive rehabilitation training; *ELISA* Enzyme-linked immunosorbent assay; *HBV* Hepatitis B virus Kaufman assessment battery for children; *RANTES* Regulated on activation, normal T cell expressed and secreted; *RDT* reaction; *Pf* Plasmodium falciparum; *SMA* Severe malarial anaemia

Boström et al*.* showed significantly lower RANTES levels in infected Dogon populations compared to uninfected Dogon, but found no difference in RANTES levels between infected and uninfected Fulani [32]. Aguilar et al*.* [29], Boström et al. [33], and Reuterswärd et al. [44] similarly reported lower RANTES levels in infected individuals compared to uninfected individuals [29]. Hojo-Souza et al*.* demonstrated lower RANTES levels in individuals with *P. vivax* infection compared to endemic controls [38]. In contrast, several studies did not find significant differences in RANTES levels between infected individuals and uninfected controls [30, 35, 39, 45, 46]. For example, Turner et al*.* found no difference in RANTES levels between infected individuals and uninfected adults [46], Jain et al*.* observed no difference between different malaria groups and healthy controls [39], and Armah e*t al.* reported no difference between children with malaria and non-malarial controls [30]. Cruz et al*.* [35, 45] found no significant difference in RANTES levels between symptomatic vivax patients and uninfected controls. Suguitan et al. [45] also found no significant difference in RANTES levels between *P. falciparum-infected* and uninfected individuals. Obeng-Aboagye et al. noted higher RANTES levels in severe malaria compared to febrile controls but no difference between uncomplicated malaria and febrile controls [11].

### RANTES levels in patients with malaria in relation to disease severity

Eight studies examined RANTES levels in patients with malaria across different complications and severities [11, 30, 34, 39, 41–43, 48]. Some studies reported no significant differences in RANTES levels between various groups. For instance, Jain et al. found no difference in RANTES levels between cerebral malaria survivors, non-survivors, and mild malaria cases [39]. Armah et al*.* observed no difference in RANTES levels between children with cerebral malaria and severe malarial anaemia [30]. Ong’echa et al*.* similarly reported no differences among patients with severe malarial anaemia, non-severe malarial anaemia, and uncomplicated malaria [42]. Obeng-Aboagye et al. revealed no variation in RANTES levels between severe and uncomplicated malaria [11].

In contrast, other studies identified significant differences in RANTES levels based on malaria severity. Were et al. observed decreasing RANTES levels with increasing severity of malarial anaemia, with the severe malarial anaemia group showing lower circulating RANTES than children with moderate and mild malarial anaemia [48]. Ochiel et al*.* demonstrated significantly lower RANTES levels in children with severe malaria compared to those with mild malaria [41]. Panda et al. found significantly lower RANTES levels in severe malaria compared to non-complicated malaria [43]. Bujarbaruah et al*.* reported significantly lower serum RANTES concentrations in severe malaria cases (15,708.92 ng/L) compared to uncomplicated malaria cases (16,147.74 ng/L) [34].

### Other findings about RANTES in patients with malaria

John et al. found that RANTES levels at 72 h after admission were significantly higher than those at the time of admission. These levels were comparable to those observed in children with uncomplicated malaria but still lower than those in community controls [15]. Hojo-Souza et al*.* demonstrated a significant increase in RANTES levels after treatment when following the same individuals [38]. Daveport et al*.* reported that RANTES levels were highest in co-infected individuals, high in individuals with malaria alone, and lowest in those exposed to both human immunodeficiency virus (HIV) and *P. falciparum* infections [36]. Vinhaes et al*.* showed significantly higher RANTES levels in symptomatic malaria compared to asymptomatic cases [47]. Noone et al*.* found no association between RANTES and *P. falciparum* parasitaemia [40]. Boivin et al*.* observed that severe malaria survivors with higher plasma RANTES levels showed better cognitive performance after receiving a computerized cognitive rehabilitation training (CCRT) intervention [31].

## Discussion

The data from the included studies provide a comprehensive view of the role of RANTES in malaria pathogenesis and severity. Several studies highlighted lower RANTES levels in patients with severe malaria compared to non-malaria controls, suggesting a potential role of RANTES in the immune response against *Plasmodium* infection. For example, Were et al*.* [48] and John et al*.* [15] found decreased RANTES levels in severe malarial anaemia and cerebral malaria, respectively, suggesting that reduced RANTES may be associated with severe disease outcomes. Conversely, studies by Jain et al*.* [39] and Armah et al*.* [30] did not find significant differences in RANTES levels between malaria patients and non-malarial controls, indicating the complexity of RANTES regulation across different patient populations and malaria presentations.

The variation in RANTES levels among different ethnic groups, as shown in studies by Boström et al., suggests that genetic or environmental factors might influence RANTES expression. Moreover, the differential RANTES response to treatment, as demonstrated by Hojo-Souza e*t al.* [38], underscores the dynamic nature of RANTES during the course of the disease and recovery. A key observation from the synthesis is the potential protective role of RANTES in uncomplicated malaria, where higher RANTES levels might contribute to better clinical outcomes and recovery, as indicated by the improved cognitive performance in severe malaria survivors with higher RANTES levels undergoing rehabilitation [31]. However, the association of low RANTES levels with thrombocytopenia, a common condition in severe malaria, points to the intricate link between platelet counts and RANTES levels. This link is supported by studies such as that of Frimpong et al*.* [37], which found significant differences in RANTES levels among children with sepsis, malaria, and febrile controls, suggesting that RANTES could serve as a biomarker for differentiating these conditions.

The immunomodulatory role of RANTES in reducing malaria pathogenesis aligns with observations that RANTES levels are lower during periods of lower malaria transmission intensity [29]. Previous studies have shown that neither serum nor cerebrospinal fluid (CSF) levels of RANTES are predictive of cerebral malaria mortality [30]. According to RCTs, individuals who survived severe malaria and had higher plasma and CSF RANTES levels after receiving rehabilitation training outperformed those who did not in terms of cognitive performance [31], implying that lower RANTES levels during acute illness are generally associated with more adverse clinical outcomes.

Low levels of RANTES in malaria patients may be explained by reduced platelet counts during acute *Plasmodium* infections [32, 49, 50]. Additionally, decreased RANTES levels could be attributed to the reduction of CD8 + T cells during *Plasmodium* infections [51, 52]. In pregnant women with acute *P. falciparum* infection, decreased RANTES levels have been reported, which is potentially associated with pregnancy-related stress [33]. According to a prior study, pregnant women with acute, uncomplicated malaria are more thrombocytopenic than non-pregnant women [53]. Studies have suggested that RANTES concentrations could differentiate between children with sepsis, clinical malaria, and febrile controls [37]. Lower RANTES levels in children with cerebral malaria have been linked to higher mortality in cases of severe malaria, even after controlling for other cytokine levels [15]. Thrombocytopenia has also been associated with decreased RANTES levels, while normal platelet counts have been found to correlate with normal RANTES levels [15]. Previous studies have suggested that RANTES concentrations directly mediate protection from severity and aid recovery in uncomplicated malaria cases by controlling the expression and modulation of monocytes, which in turn regulate the downstream effector cytokine TNF [34]. Elevated TNF levels have been correlated with disease severity [54, 55].

The mechanism linking RANTES to cerebral malaria may involve its role in mediating *Plasmodium* infection control, with impaired RANTES production potentially leading to severe malaria and increased mortality in children with cerebral malaria [15]. RANTES production occurs in both the brain and peripheral circulation, including lymphocytes, monocytes, and platelets [19]. Therefore, RANTES may have varying effects in these areas, primarily influencing regions where sequestration occurs (the brain) and having less impact in peripheral circulation, where local inflammatory mediators and cell concentrations may be less pronounced. Studies have shown decreased mRNA and protein levels of RANTES in children with severe malaria, suggesting that higher RANTES levels may offer protection against severe disease [41].

This study has some limitations. Firstly, the quantitative data from the included studies were insufficient to conduct a meta-analysis, precluding comparison of pooled mean differences in RANTES levels between participant groups. Secondly, other chemokines or cytokines involved in malaria pathogenesis may also influence RANTES levels in malaria patients. Therefore, further research on RANTES in conjunction with these molecules could provide a clearer understanding of how RANTES modulation might be leveraged for therapeutic interventions in malaria.

## Conclusion

The literature demonstrates that RANTES levels tend to be lower in infected participants compared to non-malarial controls. However, whether RANTES levels correlate with malaria severity and patient outcomes still requires further investigation, as individual studies have shown mixed results—some indicating that RANTES levels decrease with increasing malaria severity, while others show no significant difference among different severities. Future studies should prioritize longitudinal assessments of RANTES levels throughout the disease progression and recovery phases. This approach could provide a clearer understanding of how RANTES modulation might be leveraged for therapeutic interventions in malaria.

## Supplementary Information

Additional file 1

Additional file 2

Additional file 3

## Data Availability

All relevant data are within the manuscript and its Supporting Information files.
