# Supplementary material for: Association between RANTES/CCL5 levels with Plasmodium infections and malaria severity: a systematic review
Source: Malar J. 2024 Nov 9;23:335. doi: 10.1186/s12936-024-05152-1 (PMC11550525; doi:10.1186/s12936-024-05152-1)
Supplement: Supplementary file 1 — Additional file 1 [file 12936_2024_5152_MOESM1_ESM.docx]

**Table S1. Search terms**

**General keywords**

(RANTES OR CCL5 OR “RANTES Protein” OR “T-Cell RANTES Protein” OR “CCL5 Chemokine”) AND (malaria OR plasmodium OR “Plasmodium Infection“ OR “Remittent Fever“ OR “Marsh Fever“ OR Paludism)

PubMed 14 March 2024

| No. | Key concept | Search terms | Results |
| --- | --- | --- | --- |
| 1. | RANTES | RANTES[All Fields] OR CCL5[All Fields] OR “RANTES Protein”[All Fields] OR “T-Cell RANTES Protein”[All Fields] OR “CCL5 Chemokine”[All Fields] OR RANTES[MeSH Terms] OR CCL5[MeSH Terms] OR “RANTES Protein”[MeSH Terms] OR “T-Cell RANTES Protein”[MeSH Terms] OR “CCL5 Chemokine”[MeSH Terms] | 12,785 |
| 2. | Malaria | "malaria"[MeSH Terms] OR "malaria"[All Fields] OR "malarias"[All Fields] OR "malaria s"[All Fields] OR "malariae"[All Fields] OR ("plasmodium"[MeSH Terms] OR "plasmodium"[All Fields] OR "plasmodiums"[All Fields] OR "plasmodium s"[All Fields]) OR "Plasmodium Infection"[All Fields] OR "Remittent Fever"[All Fields] OR "Marsh Fever"[All Fields] OR ("malaria"[MeSH Terms] OR "malaria"[All Fields] OR "paludism"[All Fields]) | 126,457 |
| 3. | #1 AND #2 | (RANTES[All Fields] OR CCL5[All Fields] OR “RANTES Protein”[All Fields] OR “T-Cell RANTES Protein”[All Fields] OR “CCL5 Chemokine”[All Fields] OR RANTES[MeSH Terms] OR CCL5[MeSH Terms] OR “RANTES Protein”[MeSH Terms] OR “T-Cell RANTES Protein”[MeSH Terms] OR “CCL5 Chemokine”[MeSH Terms]) AND ("malaria"[MeSH Terms] OR "malaria"[All Fields] OR "malarias"[All Fields] OR "malaria s"[All Fields] OR "malariae"[All Fields] OR ("plasmodium"[MeSH Terms] OR "plasmodium"[All Fields] OR "plasmodiums"[All Fields] OR "plasmodium s"[All Fields]) OR "Plasmodium Infection"[All Fields] OR "Remittent Fever"[All Fields] OR "Marsh Fever"[All Fields] OR ("malaria"[MeSH Terms] OR "malaria"[All Fields] OR "paludism"[All Fields])) | 85 |

Embase 14 March 2024

| No. | Key concept | Search terms | Results |
| --- | --- | --- | --- |
| 1. | RANTES | RANTES:ti,ab,kw,de OR CCL5:ti,ab,kw,de OR “RANTES Protein”:ti,ab,kw,de OR “T-Cell RANTES Protein”:ti,ab,kw,de OR “CCL5 Chemokine”:ti,ab,kw,de OR RANTES/exp OR CCL5/exp OR “RANTES Protein”/exp OR “T-Cell RANTES Protein”/exp OR “CCL5 Chemokine”/exp | 30,686 |
| 2. | Malaria | malaria:ti,ab,kw,de OR plasmodium:ti,ab,kw,de OR ‘Remittent Fever’:ti,ab,kw,de OR ‘Marsh Fever’:ti,ab,kw,de OR Paludism:ti,ab,kw,de OR malaria/exp | 161,383 |
| 3. | 1 AND 2 | (RANTES:ti,ab,kw,de OR CCL5:ti,ab,kw,de OR “RANTES Protein”:ti,ab,kw,de OR “T-Cell RANTES Protein”:ti,ab,kw,de OR “CCL5 Chemokine”:ti,ab,kw,de OR RANTES/exp OR CCL5/exp OR “RANTES Protein”/exp OR “T-Cell RANTES Protein”/exp OR “CCL5 Chemokine”/exp) AND (malaria:ti,ab,kw,de OR plasmodium:ti,ab,kw,de OR ‘Remittent Fever’:ti,ab,kw,de OR ‘Marsh Fever’:ti,ab,kw,de OR Paludism:ti,ab,kw,de OR malaria/exp) | 227 |

Scopus 14 March 2024

| No. | Key concept | Search terms | Results |
| --- | --- | --- | --- |
| 1. | RANTES | TITLE-ABS-KEY ( rantes OR ccl5 OR "rantes protein" OR "t-cell rantes protein" OR "ccl5 chemokine" ) | 25,064 |
| 2. | Malaria | TITLE-ABS-KEY ( malaria OR plasmodium OR "plasmodium infection" OR "remittent fever" OR "marsh fever" OR paludism ) | 162,636 |
| 3. | 1 AND 2 | TITLE-ABS-KEY ( rantes OR ccl5 OR "RANTES Protein" OR "T-Cell RANTES Protein" OR "CCL5 Chemokine" ) ) AND ( TITLE-ABS-KEY ( malaria OR plasmodium OR "Plasmodium Infection" OR "Remittent Fever" OR "Marsh Fever" OR paludism ) | 207 |

MEDLINE 14 March 2024

| No. | Key concept | Search terms | Results |
| --- | --- | --- | --- |
| 1. | RANTES AND Malaria | (RANTES OR CCL5 OR “RANTES Protein” OR “T-Cell RANTES Protein” OR “CCL5 Chemokine”) AND (malaria OR plasmodium OR “Plasmodium Infection“ OR “Remittent Fever“ OR “Marsh Fever“ OR Paludism) | 82 |

Ovid 14 March 2024

| No. | Key concept | Search terms | Results |
| --- | --- | --- | --- |
| 1. | RANTES AND Malaria | (RANTES OR CCL5 OR “RANTES Protein” OR “T-Cell RANTES Protein” OR “CCL5 Chemokine”) AND (malaria OR plasmodium OR “Plasmodium Infection“ OR “Remittent Fever“ OR “Marsh Fever“ OR Paludism)  Filter: limit to (ovid full text available and articles with abstracts and original articles) | 414 |

ProQuest 14 March 2024

| No. | Key concept | Search terms | Results |
| --- | --- | --- | --- |
| 1. | RANTES AND Malaria | (RANTES OR CCL5 OR “RANTES Protein” OR “T-Cell RANTES Protein” OR “CCL5 Chemokine”) AND (malaria OR plasmodium OR “Plasmodium Infection“ OR “Remittent Fever“ OR “Marsh Fever“ OR Paludism) | 473 |

Google Scholar 14 March 2024

| No. | Key concept | Search terms | Results |
| --- | --- | --- | --- |
| 1. | RANTES AND Malaria | RANTES and malaria | The first 200 articles |
